# Comparison of Individualized Rescue Luteal Phase Support Strategies with Vaginal and Combined Vaginal & Subcutaneous Progesterone Administration in Artificial Frozen-Thawed Blastocyst Embryo Transfer Cycles Based on Serum Progesterone levels

**DOI:** 10.3389/fendo.2024.1503008

**Published:** 2025-01-17

**Authors:** Secil Irem Arik Alpcetin, Onur Ince, Bengisu Akcay, Munire Funda Cevher Akdulum, Erhan Demirdag, Ahmet Erdem, Mehmet Erdem

**Affiliations:** ^1^ Department of Obstetrics and Gynecology, Gazi University, Ankara, Türkiye; ^2^ Department of Obstetrics and Gynecology, Hacettepe University, Ankara, Türkiye; ^3^ NovaArt Reproductive Health and Fertility Center, Ankara, Türkiye

**Keywords:** infertility, frozen embryo replacement cycle, progesterone, luteal phase support (LPS), assisted reproductive techniques

## Abstract

**Objectives:**

Hormone replacement therapy (HRT) frozen embryo transfer (FET) cycles are common in assisted reproductive techniques. As the corpus luteum is absent in these cycles, luteal phase support is provided by administering progesterone (P4) through transvaginal, parenteral, or oral routes. Low serum levels of P4 (below 9-10 ng/mL) on the day before embryo transfer (ET) have been associated with unfavorable cycle outcomes. The aim of this study is to investigate whether individualizing luteal support through rescue protocols in patients with low serum P4 levels improves pregnancy outcomes in HRT-FET cycles.

**Material and method:**

This retrospective, single-center cohort analysis includes 1257 cycles involving 942 patients undergoing HRT-FET. Starting in 2019, we have assessed P4 levels before ET day and adjusted MVP doses when P4 levels were <10 ng/mL. In 2021, subcutaneous (SC) P4 was routinely added alongside MVP, with SC doses increased if P4 levels were <10 ng/mL. In this study, Groups 1 and 2 received MVP for luteal support, while Groups 3 and 4 received additional SC progesterone. For patients with P levels below the cut-off level (10 ng/mL) in Groups 2 and 4, the P dose was doubled through a rescue protocol.

**Results:**

In the MVP and MVP plus SC groups, 15.8% and 8.9% of the cycles had P4 levels <10 ng/mL, respectively. Ongoing pregnancy rates (OPR) and clinical pregnancy rates (CPR) did not differ between study groups. Regression analysis with a mixed model revealed that age, endometrial thickness, and estradiol levels were confounding factors as well as independent predictors of ongoing pregnancy rates (p<0.05). Pairwise regression analysis revealed no significant differences in pregnancy rates between the groups (p>0.05).

**Conclusion:**

Individualizing luteal phase support based on serum P4 levels on the day of ET in FET cycles with HRT may enhance pregnancy outcomes by either doubling the vaginal dose or increasing the SC dose during MVP plus SC administration. The implemented rescue protocol allowed patients with low progesterone levels to achieve pregnancy outcomes similar to those with higher progesterone levels.

## Introduction

Frozen embryo transfer (FET) has been a widely used technique in assisted reproductive technology (ART) practice (1). According to the 2019 Society for Assisted Reproductive Technology (SART) report, approximately 41% of all embryo transfers were FET cycles in the US (2). In recent years, the use of FET cycles has gradually increased due to better cryopreservation techniques, and this approach allows the use of the freeze-all strategy, preimplantation genetic testing, protection against ovarian hyperstimulation syndrome (OHSS), and is advantageous in various situations, such as endometrial abnormalities and management premature progesterone elevation before triggering ovulation in fresh cycles (3).

Progesterone (P_4_) is essential for preparing the endometrium for implantation and plays a crucial role in the success of Fresh cycle and FET cycles. In hormone replacement (HRT) FET cycles, the corpus luteum is absent, and the luteal phase support (LPS) is dependent on P_4_ supplementation for initiating and maintaining the secretory endometrium and pregnancy with different routes, including transvaginal, intramuscular (IM), subcutaneous (SC), or oral administration (4). For that reason, the role of P_4_ has been widely studied in HRT FET cycles: especially measuring the levels of P_4_ on the day of embryo transfer (ET) has been suggested as a keynote to improve the success rates of FET.

There is growing evidence to suggest that the serum level of P4 before ET may have an impact on the outcome of FET (5, 6). Specifically, recent studies on the threshold levels of P_4_ before the day of FET have shown that low levels of P_4_ (below 9-10 ng/ml) are associated with worse pregnancy outcomes. These studies reported favorable LBRs in higher P_4_ levels compared to lower levels (7–10). In contrast to these findings, LBRs were comparable when P_4_ levels of 10 ng/ml were used as a threshold (11). Thus, the impact of the serum level of P_4_ before ET and routes of P_4_ administration on pregnancy outcomes in patients undergoing FET is still insufficiently explored in the literature.

Studies on luteal support in FET cycles mostly focus on vaginally administered progesterone. Research on non-vaginal routes of luteal phase support, as well as dosage and progesterone threshold values, are insufficient (12). Studies on subcutaneous progesterone mainly involve the addition of subcutaneous progesterone as a rescue treatment for patients using vaginal progesterone who have progesterone levels below the threshold on the day of embryo transfer (9, 13, 14).

In HRT-FET cycles using subcutaneous progesterone, it is not yet clear whether the necessity for rescue is eliminated or whether the requirement for measuring progesterone levels on the day of embryo transfer is no longer needed. For this reason, we aimed to evaluate the impact of serum P_4_ level on the day of ET via personalized progesterone supplementation approach by comparing the effectiveness of vaginal administration alone versus a combination of vaginal and subcutaneous routes.

## Materials and methods

This retrospective, single-center (NovaART IVF Center) cohort study includes 1257 cycles involving 942 patients undergoing FET from January 2019 to December 2022. The study was approved by the Ethics Committee of Gazi University Faculty of Medicine (approval number: 2023/918).

### Study design

The measurement of serum P_4_ levels on the day of ET and the individualization of the luteal phase through rescue P_4_ supplementation with an additional MVP dose when P_4_ levels are below 10 ng/ml have become an integral part of our FET protocols. If the progesterone level is below 10 ng/ml on the day of embryo transfer, we implemented an individualized strategy by doubling the MVP dose. The cut-off level of 10 ng/ml was determined based on the findings of published prospective studies (6, 11, 15, 16). We have recently revised our protocol after 2021 by adding subcutaneous progesterone (SCP) to MVP for all patients with an additional SCP dose as a rescue if the P4 level was below 10 ng/ml on the day of ET. (17).

### Study groups

The study population was divided into four groups. The group that started on vaginal progesterone showed a higher proportion of individuals with low progesterone levels before ET compared to those who started on SCP. Additionally, SCP administration prior to transfer has become common practice in all cycles in recent years, due to the higher progesterone levels observed in cycles where SCP is used as demonstrated in recent studies.

Group 1: Luteal MVP with normal P_4_ levels (247 patients/326 cycles).

Group 2: Luteal MVP with low P_4_ levels (individualized rescue protocol by doubling the MVP dose) (155 patients/198 cycles).

Group 3: Luteal SCP in addition to vaginal P_4_ with normal P_4_ levels (462 patients/621 cycles).

Group 4: Luteal SCP in addition to vaginal P_4_ with low P_4_ levels (rescue by doubling the SCP) (78 patients/112 cycles).

### Eligibility criteria

Patients who completed FET cycles, were between the ages of 20-45, had a BMI of less than 40 kg/m², and had no systemic diseases were included in the study. All patients utilized their own embryos. One or two good quality blastocysts were transferred to patients with normal endometrial pattern (triple layer) and thickness (> 6.5 mm). Patients with non-surgically treated major uterine anomalies, or intrauterine lesions such as polyps, fibroids, or hydrosalpinx were excluded from the study. We also excluded cycles canceled in patients with P_4_ rise before luteal support, uterine bleeding, endometrial abnormalities, including thin or echogenic endometrium, uterine peristalsis diagnosed by real time ultrasonography, fluid accumulation in uterine cavity, low estradiol serum levels after exogenous estrogen administration and other reasons (COVID infection, patient requested etc.).

### HRT protocol

In HRT cycles, 2 mg oral estradiol (E_2_) (Estrofem, Novo Nordisk, Turkey) was started three times daily on the 3^rd^ day of the menstrual cycle. The patients were evaluated by serum E_2_ measurement and transvaginal ultrasound on day 7 to determine endometrial thickness. Estradiol dose was adjusted according to the thickness and serum E_2_ levels. In Group 1 and 2, when the endometrial thickness was ≥ 6.5 mm and E2 level was >150 pg/ml, vaginal micronized progesterone (Progestan, Koçak, Farma, Turkey) was administered as 200 mg MVP capsules twice daily. On the day of ET, patients with a P4 level ≥ 10 pg/ml continued to receive the same doses of exogenous vaginal P4 (*Group 1*). However, in patients with a P4 level less than 10 pg/mL, the dose of MVP was doubled and administered twice daily for LPS (*Group 2*). In Group 3 and 4, LPS consisted of 200 mg MVP capsules (Progestan, Koçak Farma, Turkey) twice daily and 25 mg SCP (Progestan Dex, Koçak Farma, Turkey) once daily. The same dose of exogenous P_4_ was used in patients with serum P_4_ level ≥10 pg/ml (*Group 3*). The patients with P4 level <10 pg/ml had their SCP dose doubled for LPS (*Group 4*).

ET was performed on the 6th day of P4 supplementation. Estradiol and P_4_ supplementation continued until the end of the 9^th^ week of pregnancy.

### Hormone measurement

Serum P_4_ concentrations were measured by an electrochemiluminescence immunoassay (Cobas Elecsys Progesterone III, Roche Diagnostics GmbH, Germany). The intra-assay coefficient of variation was 2.4%, and the interassay coefficient of variation was 3.9%. Serum P4 concentrations were measured 4-6 hours after the last vaginal progesterone dose in the morning and 12-16 hours after the last SC dose.

### Vitrification and embryo transfer

The vitrification technique was used for cryopreservation in all FET cycles. Fertilization of all embryos was achieved using the Intracytoplasmic Sperm Injection (ICSI) method. Embryos were thawed on the day of ET.

The morphology and cell count of the embryos were assessed on the day of transfer to ascertain their quality based on the Gardner and Schoolcraft embryo grading system, with only embryos graded as ≥ 3BB being selected for transfer (18). One or two embryos were transferred under transabdominal ultrasound guidance using a flexible catheter (Wallace; Irvine Scientific, Santa Ana, CA).

### Outcome measures

The primary outcome measure was ongoing pregnancy rate (OPR) among different LPS groups. The secondary outcome measure was the clinical pregnancy rate (CPR).

Clinical pregnancy was defined as the presence of at least one gestational sac observed on ultrasound. Biochemical abortion was defined as pregnancy loss before the gestational sac was identified on ultrasound. Miscarriage was defined as the loss of a non-viable fetus before 20 weeks of gestation. Ongoing pregnancy was defined as a pregnancy beyond 12 weeks.

### Statistical analysis

Data were analyzed by Statistical Package for Social Sciences (SPSS, version 26.0, Statistics, 2020, Chicago, IBM, USA) and R Version 3.6.1 (https://www.r-project.org/). The distribution of the analyzed data was assessed. When the data did not follow a normal distribution, the non-parametric Kruskal-Wallis test was employed to compare differences between groups. Bonferroni correction was applied for non-normally distributed data to account for multiple comparisons and minimize the risk of Type I errors (false positives). The chi-square test or Fisher’s exact test was used to compare categorical variables. Continuous variables were presented as median and interquartile range (IQR), while percentages were used for categorical variables. Statistical significance was set at p<0.05.

A *post hoc* power analysis was conducted using R Version 3.6.1 (https://www.r-project.org/) to evaluate the power of the Chi-squared test used to assess differences in pregnancy rates across four groups with sample sizes of 326, 198, 621, and 112, respectively. An effect size (Cohen’s w) of 0.1, representing a relatively small effect size and indicating minimal clinical pregnancy differences, was used along with a significance level of 0.05 for a two-sided test. The analysis yielded a power of 86%, indicating an 86% probability of detecting a statistically significant difference in pregnancy rates among the groups.

A mixed-effects logistic regression model was used to predict the OPR by protocol type, adjusting for age, BMI, duration of infertility, endometrial thickness, E2 levels (included in the model per 100 pg/ml increase), and the number of transferred embryos, with a random effect added to account for repeated cycles from the same patients. Pairwise comparisons between different protocol types were adjusted using the Tukey method for the comparison of four estimated values. The estimated ongoing pregnancy probabilities, adjusted for confounders and random effects, were presented for the mean values of covariates and the reference values of cofactors.

## Results

In this study, 1386 cycles from 942 patients were evaluated. 129 cycles were canceled due to various reasons, including P4 rise before luteal support (n=54), uterine bleeding (n=12), endometrial abnormalities such as thin endometrium (n=11), endometrial polyp (n=7), endometrial peristalsis (n=12), echogenic endometrium (n=3), fluid accumulation in the uterine cavity (n=6), low serum E2 level before P4 supplementation (n=2), and other reasons (n=16), such as COVID infection and patient requests. The remaining 1257 cycles were used for statistical evaluation.

### Demographic data

A comparison of demographic features, and clinical findings of the groups is presented in **Table 1**. When demographic data were compared among the groups, significant differences were found only in age, BMI, endometrial thickness, and the number of transferred embryos. BMI of the patients in Group 1 and 2 was significantly lower than that of Groups 3 and 4 (p<0.05). The age of the patients in Groups 2 was significantly lower than in Groups 1 and 3. The mean endometrial thickness was higher in Groups 2 and 4 than in Group 3. The mean serum E_2_ levels prior to P_4_ supplementation were higher in Group 3 than in Group 1 and 4. The proportion of cycles with two embryos transferred was lower in Group 3 than in the other groups (p<0.05).

**Table 1:** Comparison of demographic and clinical outcomes between whole study populations.

	Total Population	Group 1 Only MVP with normal P_4_	Group 2 MVP with low P_4_	Group 3: MVP + SCP with normal P_4_	Group 4: MVP + SCP with low P_4_	*p-value*
Age (years)	32 (28-36)	32 (28-37)^a^	31 (28-34) ^b^	32 (29-36) ^a^	32 (29-35)^ab^	**0.047**
BMI (kg/m^2^)	23 (22-26)	22 (22-25)^a^	22 (22-25) ^a^	24 (22-27,9)^b^	23,3 (22-26)^b^	**<0.001**
Duration of infertility	4 (2-7)	4 (2-7)	4 (2,5-7)	4 (2-7)	5 (3-8)	0. 408
Endometrial thickness before P_4_ supplement (mm)	9.8 (8.7-11.0)	9.8 (8.5-11.0)^ab^	10.0 (9-11)^a^	9.5 (8.5-11.0)^b^	10 (9-11)^a^	**<0.001**
E_2_ levels before P_4_ supplement (pg/ml)	228 (183-289)	210 (172-282)^a^	234 (179-291)^ab^	236 (192-295)^b^	218 (170-269)^a^	**<0.001**
Number of single embryos transferred: (%)	43%	32.8%	28.3%	54.1%	37.5%	**<0.001**
P_4_ levels on the day of ET (ng/ml)	14 (10-18)	13.7 (11.8-17.3)^a^	7.6 (6.4-8.8)^b^	17.25 (14.1-22.0)^c^	7.8 (6.4-9.0)^b^	**<0.001**
CPR, n (%)	686 (54.6)	186 (57.1)	105 (53.0)	327 (52.7)	68 (60.7)	0.305
OPR, n (%)	525 (41.8)	138 (42.3)	82 (41.4)	248 (39.9)	57 (50.9)	0.192
Multiple pregnancy rate, n (%)	66 (7.3)	19 (5.8)	13 (6.6)	25 (8.9)	9 (9)	0.123
Miscarriage rate, n (%)	157 (12.5)	48 (14.7)	23 (11.6)	76 (12.2)	10 (8.9)	0.397
Estimated Ongoing Prengancy Probabilty with adjustment for confounding variables % [%95 CI]	-	43% [34-52]	42% [30-54]	44% [39-50]	50% [35-65]	-

Data are presented as median (interquartile range: 25th-75th percentile), numbers, and percentages.

BMI, Body Mass Index; E2, Estradiol; P, Progesterone; MVP, Micronized Vaginal Progesterone; SCP, Subcutaneous Progesterone; CPR, Clinical Pregnancy Rate; OPR, Ongoing Pregnancy Rate;

[%95 CI], Confidence Intervals.

Superscript letters (a, b, c) indicate compact display of statistically significant differences between groups p-value of <0.05 was considered statistically significant. Bold values indicate statistical significance with a p-value < 0.05.

### Progesterone levels before ET

In Groups 2 and 4, 15.8% and 8.9% of the cycles had low P4 levels, respectively. The mean progesterone levels on the day of ET were 7.4 ± 1.7 ng/mL and 7.5 ± 1.9 ng/mL in Groups 2 and 4, respectively, compared to 15.5 ± 6.1 ng/mL in Group 1 and 19.7 ± 9.2 ng/mL in Group 3 (**Table 1**) (p<0.05). Among the groups with low progesterone levels, no significant differences were observed between Group 2 and Group 4.

### Pregnancy rates

In study groups, ß-hCG was positive in 65.2% of all study cycles; 5.5% and 12.5% of the cycles ended with biochemical abortion and miscarriage, respectively. Sixty-six (7.3%) patients had twin pregnancies. The overall CPR and OPR per cycle were 54.6% and 41.8%, respectively, in the study population. When the study groups were compared among each other regarding pregnancy outcome, no significant differences were found in CPR and OPR between groups (**Table 1**).

When evaluating the pregnancy rates of the groups compared to each other, we adjusted for confounders and calculated the estimated pregnancy rates of the other groups relative to the reference group. When Group 1 was used as the reference, the estimated pregnancy outcomes for the other groups, expressed as odds ratios (OR) with 95% confidence intervals (CI), were as follows: Group 2: OR = 0.95 [95% CI: 0.52-1.74], Group 3: OR = 1.06 [95% CI: 0.68-1.65], and Group 4: OR = 1.35 [95% CI: 0.66-2.77].

### Clinical factors affecting outcome

**Figure 1:**
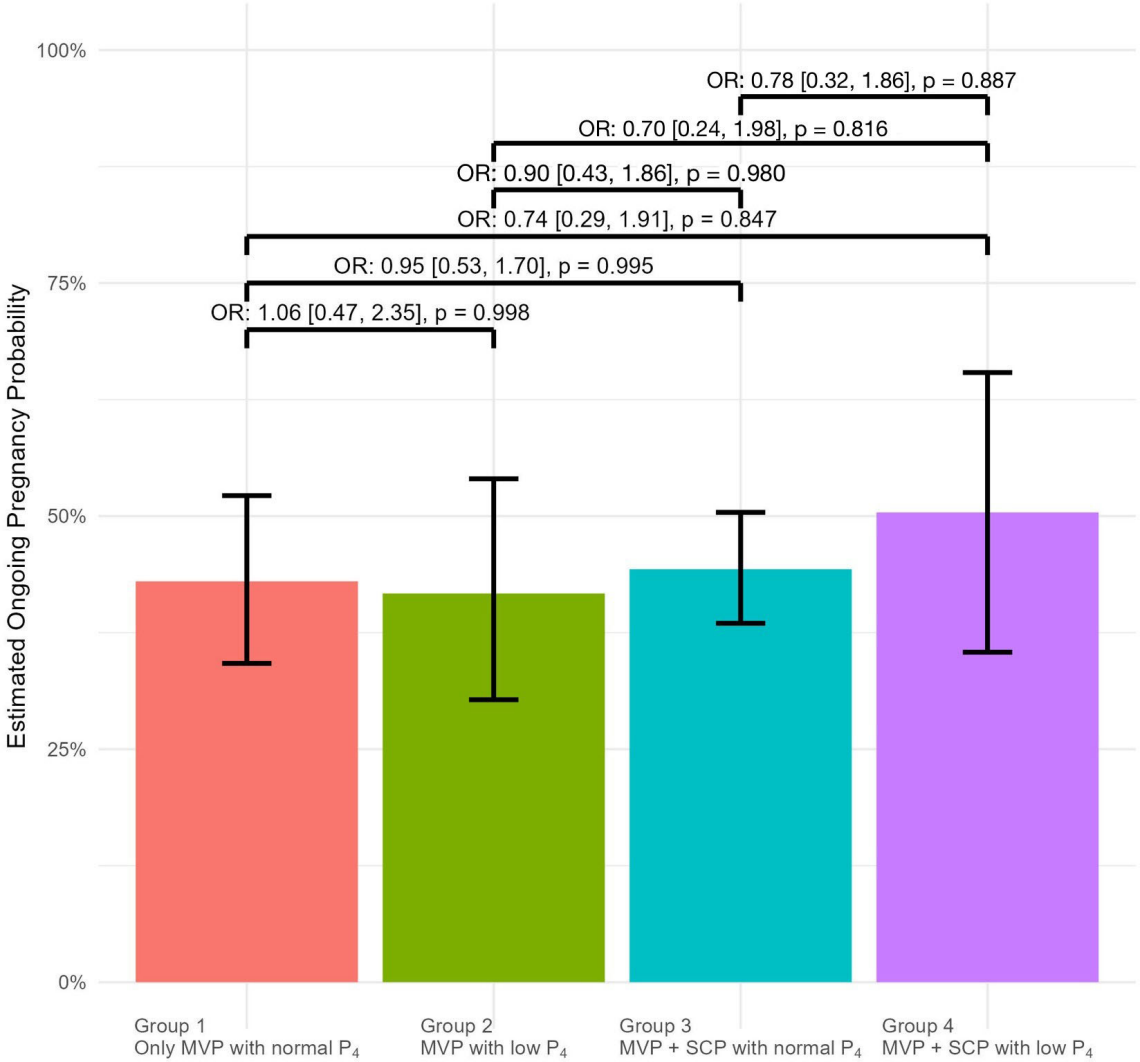
The odds ratios (ORs) for ongoing pregnancy outcomes are displayed above the brackets for pairwise comparisons between the groups on the x-axis. In each comparison, the group appearing earlier on the x-axis was used as the reference. OR[%95 CI] : Odds Ratio with %95 Confidence Intervals.

A mixed regression model was used to identify independent variables predicting ongoing pregnancy. In the mixed regression model, variables were adjusted for age, BMI, duration of infertility, E2 levels, and endometrial thickness, taking into account the number of repeated cycles. The results of the multivariate and univariate regression analyses for variables affecting pregnancy outcomes were presented in **Table 2**. Age, endometrial thickness, and estradiol levels were confounding factors and independent predictors of ongoing pregnancy rates. The analysis revealed that BMI, duration of infertility, and the number of transferred embryos did not have a significant impact on pregnancy outcomes. Additionally, when assessing the impact of protocol types on pregnancy outcomes, pairwise analysis of protocol types revealed no significant differences in pregnancy rates between groups compared to the reference group, as presented in **Figure 1**.

**Table 2:** Regression analyzes to predict ongoing pregnancy rate.

	Effect on Ongoing Pregnancy Rate			
	Univariate		Multivariate	
	OR [%95 CI]	p value	OR [%95 CI]	p value
Age	0.94 [0.908-0.976]	0.001	0.94 [0.908-0.980]	0.002
BMI	0.97 [0.937-1.013]	0.197	0.97 [0.930-1.009]	0.135
Duration of infertility	0.98 [0.942-1.026]	0.453	1.01 [0.964-1.056]	0.692
Endometrial thickness	1.13 [1.0266-1.243]	0.012	1.11 [1.010-1.228]	0.029
E_2_ levels before P_4_ supplement (per 100 pg/ml increase)	0.80 [0.666-0.971	0.023	0.82 [0.673-0.992]	0.041
Transferred Embryos Number	0.95 [0.668-1.350]	0.775	1.05 [0.724-1.532]	0.783
Group 1 Only MVP with normal P_4_	1 [Ref]	–	1 [Ref]	–
Group 2 MVP with low P_4_	1.01[0.554-0.845]	0.970	0.95 [0.514-1.742]	0.861
Group 3: MVP + SCP with normal P_4_	1.02 [0.665-1.577]	0.912	1.06 [0.677-1.648]	0.807
Group 4: MVP + SCP with low P_4_	1.39 [0.690-2.806]	0.354	1.35 [0.656-2.773]	0.414

Data are presented as Odds Ratio, Confidence Interval, and p-value OR[%95 CI], Odds Ratio with %95 Confidence Intervals.

BMI, Body Mass Index; E2, Estradiol; P, Progesterone; MVP, Micronized Vaginal Progesterone; SCP, Subcutaneous Progesterone. Bold values indicate statistical significance with a p-value < 0.05.

After adjusting for confounding variables such as age, BMI, duration of infertility, endometrial thickness, and treatment group, the estimated pregnancy rates are Group 1, 43%; Group 2, 42%; Group 3, 44%; Group 4, 50% respectively.

## Discussion

The findings from this study support the impact of low serum progesterone levels before embryo transfer on pregnancy outcomes in HRT FET cycles, as previously demonstrated in prospective studies (5–7, 12, 15, 19). An analysis of our data indicates that individualizing luteal phase support based on serum P4 levels on the day of ET in FET cycles with HRT may improve pregnancy outcomes, particularly in cases of low P4 levels. Commonly used doses range from 400 to 800 mg, but there is no standardized dose for luteal support in FET cycles (16, 20). Although a recent study by Devine et al. showed that a daily dose of 400 mg was ineffective compared to daily MVP plus IMP or IMP alone, the results of our current study clearly indicate that individualizing the daily MVP dose to 400–800 mg in patients with low P on the day of ET may help rescue the cycle and improve pregnancy outcomes. (17) This individualization can also be achieved by doubling the SCP dose during MVP plus SC administration. Moreover, it was revealed that endometrial thickness and E2 levels before P4 supplementation were significant variables predicting ongoing pregnancy, in addition to age.

Clinical trials have demonstrated that serum P_4_ level is an indicator of the bioactivity of exogenous P_4_ when using MVP (6, 7). In parenteral P_4_ administration, the endometrial P content is physiological, despite elevated circulating levels. Through the vaginal route, endometrial P levels could be subphysiological as a result of poor vaginal absorption or limited uterine first pass. In HRT-FET cycles, the myometrial levels of progesterone may be insufficient which might hypothetically lead to ineffective suppression of subendometrial uterine contractions and a diminished impact on the immune system. Indeed, serum progesterone levels demonstrated an inverse correlation with uterine contractions, and IM route notably decreased the frequency of such contractions on the day of embryo transfer (21–23). Some other studies reported that the IM route does not reduce the subendometrial activity compared to vaginal progesterone administration (24).

For those reasons, it is expected that the pregnancy outcomes of patients using the parenteral route would be better than those with vaginal dose increase, which might be due to the limited bioavailability of micronized vaginal progesterone or systemic effect of P_4_ on parenteral use (25). On the other hand, some studies have concluded that there is no difference in pregnancy outcomes between progesterone administration routes (26–28). Due to these considerations, there has been thorough investigation into both the dosage and administration route of progesterone in HRT FET cycles. Preliminary data indicate a correlation between progesterone levels on the day of embryo transfer and pregnancy outcomes in HRT-FET cycles (6, 8, 15, 29). Furthermore, in a prospective study Labarta et al. reported that an intervention should be required in patients with low P_4_ levels (7). Therefore, diversifying progesterone uptake routes and increasing uptake doses became a basic approach to improve outcomes in patients with low P4 levels, particularly for those with different P4 cut-off levels in HRT-FET cycles (6, 11, 15, 30). To our knowledge, rescue of the luteal support by increasing the vaginal dose of P_4_ in HRT-FET cycles has not been evaluated before. Our results indicated for the first time that increasing vaginal P4 by increasing the MVP dose in patients with low P4 on the day of ET rescued the HRT-FET cycles, with similar pregnancy outcomes to the subcutaneous groups. This might show that low pregnancy rates in the MVP group are due to inadequate dose rather than limited absorption (31). A previous study has also demonstrated that increasing doses of vaginal P_4_ can reduce the miscarriage rate and increase the pregnancy rate (32).

Recent studies have indicated that from 30% to 50% of women have suboptimal P_4_ serum levels before the embryo transfer, leading to compromised outcomes unless rescue of luteal support is undertaken (7, 15). In our study, a relatively lower rate of cycles with low P4 levels in the MVP and MVP plus SCP groups (15.8% and 8.9%, respectively) was observed compared to previous studies. The rate of low P levels was also lower in the MVP plus SC cycles, which indicates the efficacy of parenteral P for luteal support. However, luteal P4 monitoring and a dose increase are still necessary in the parenteral subcutaneous route, as 8% of the patients in this group still have low P levels on the day of ET.

The pregnancy outcomes of patients with parenteral rescue dose increases, either by IM or SC in addition to MVP, were better than those without rescue in patients with low P4 (14, 33, 34).

In our study, all patients with low progesterone levels received rescue treatment either with MVP or SCP. We found that pregnancy rates in the rescue groups were comparable to those in the groups with progesterone levels above the cut-off threshold. In other words, it can be concluded that the rescue protocol successfully salvaged the cycles with low P Subcutaneous progesterone appears to be an effective alternative to vaginal progesterone in patients undergoing FET when SC progesterone (50 mg/day) and vaginal progesterone gel (180 mg/day) are used (35). Two studies have demonstrated a positive effect of adding 25 mg of SCP to MVP in a single injection to patients with low serum P_4_ levels (9, 13). Devine et al. in a three arm prospective study, have shown a decreased OPR in patients with vaginal-only P_4_ replacement for FET cycles as compared to MVP plus IM progesterone every three day and daily IM progesterone groups (19). In concordance with our study, these findings indicate that patients receiving luteal support with only vaginal P4 need a dose increment in case of low P4, or the use of parenteral P4 after luteal P4 measurement. However, in contradiction to current prospective data, one recent research found that the addition of daily subcutaneous P_4_ did not improve pregnancy outcomes in HRT-FET cycles with euploid embryos (9).

In our study, we used a cut-off level of 10 ng/mL. Melo et al. in a meta-analysis compared CPR, OPR, LBR, and miscarriage rates regarding the cut-off values. It was determined that serum progesterone levels higher than the cut off 10 ng/mL were associated with better treatment outcomes on average and the increase in pregnancy rates were consistent until the serum level of 30 ng/ml (36).

The main strength of our study is that we evaluated a large sample of patients with either treatment approaches including MVP and MVP plus subcutaneous routes. Another strength is the comparison of vaginal dose increase in HRT-FET cycles in a single center, where there was no similar study in the literature. The primary limitations of this study include its retrospective design, reliance on predefined cut-off values for progesterone levels, and the possibility of bias from other confounding factors. Including multiple cycles in the study may have led to the inclusion of some patients with recurrent implantation failure, although the existence of recurrent implantation failure itself has been a highly debated topic in recent times (37, 38). Among the limitations associated with the retrospective design of the study, protocol selection was limited to a year-based preference only, constrained by the updates in the literature. Clinicians did not select any specific protocols beyond those that varied by year, nor did they opt for protocols tailored to specific situations. However, multivariate analysis was conducted to mitigate bias from confounding variables, particularly across multiple cycles involving the same patients. The differences in age and endometrial thickness have been attributed to the large sample size of the study. We did not perform a secondary measurement of serum progesterone levels after dose escalation to confirm whether levels increased following the additional administration. Ozcan et al. found that 83% of patients with low serum P_4_ concentrations on ET day reached an adequate progesterone concentration with rescue SCP treatment and 90% of pregnancies occurred in patients who reached adequate serum progesterone concentrations with daily rescue SCP treatment (39).

While our study protocols showed similar pregnancy outcomes, a non-inferiority trial would be necessary to conclusively determine that they produce identical results.

## Conclusion

In conclusion, our data demonstrate the positive impact of measuring P4 on the day of ET for improving pregnancy outcomes by enabling individualized progesterone dosing through either vaginal or parenteral route adjustments. Our findings also suggest that doubling the dose of vaginal progesterone is equally effective to the parenteral route. Future considerations should focus on determining the precise progesterone cut-off level on the day of embryo transfer, evaluating the need for and cost-effectiveness of retesting progesterone levels after dose adjustments, and examining the influence of alternative administration routes, notably oral progesterone, on pregnancy outcomes in artificial cycles.

## Data Availability

The original contributions presented in the study are included in the article/supplementary material. Further inquiries can be directed to the corresponding author.
